# Efficacy and safety of Maxing Shigan Decoction in the treatment of chronic obstructive pulmonary disease: A protocol for a systematic review and meta-analysis

**DOI:** 10.1097/MD.0000000000023284

**Published:** 2020-12-04

**Authors:** Jinyun Chen, Chunrong Wang, Min Xiong, Qilin Shen

**Affiliations:** a College of basic medicine, Chengdu university of Traditional Chinese Medicine, Chengdu; b College of basic medicine, Mianyang traditional Chinese medicine hospital, Mianyang, Sichuan, China.

**Keywords:** chronic obstructive pulmonary disease, Maxing Shigan Decoction, randomized controlled trial, systematic review

## Abstract

**Background:**

Chronic Obstructive Pulmonary Disease (COPD) is currently the fourth leading cause of death in the world but is projected to be the 3rd leading cause of death by 2030. Chronic obstructive pulmonary disease is an important public health challenge, which can be prevented and treated. COPD is an important public health challenge, both preventable and treatable. In China, Maxing Shigan Decoction (MSD) has been used as a traditional Chinese medicine compound for the treatment of respiratory diseases for thousands of years. In order to evaluate the efficacy and safety of MSD in the treatment of COPD, we need to conduct meta-analysis and systematic reviews.

**Methods:**

The data comes from 7 publicly published databases, including: PubMed, The Cochrane Central Register of Controlled Trials (CENTRAL), EMbase, China National Knowledge Infrastructure (CNKI), Chinese Biomedical Database(CBM), VIP Database, and Wanfang database. We will include randomized controlled trials (RCTs) to evaluate the effectiveness and safety of MSD in the treatment of COPD. Result measurement indicators include: TCM syndrome scores, lung function indicators, serum inflammatory factors, blood gas indicators, adverse reactions. RevMan 5.0 will be used for meta-analysis.

**Results:**

This study will provide high-quality evidence for the effectiveness and safety of traditional Chinese medicine therapy for COPD.

**Conclusion:**

The results of this study will help us determine whether MSD can effectively treat COPD.

**Ethics and dissemination:**

All analyses in this study are based on previously published research, so this study does not require ethical approval or patient consent. We will disseminate our findings electronically or in print by publishing results in peer-reviewed journals.

**OSF registration number:**

DOI 10.17605/OSF.IO/H5UNB.

## 1 Introduction

COPD is the main cause of chronic morbidity and mortality worldwide. As a common, preventable, and treatable disease, COPD is characterized by persistent respiratory symptoms and airflow restrictions, which are due to airways and/ Or alveolar abnormalities, usually caused by exposure to harmful particles or gases, and are affected by host factors including abnormal lung development.^[1]^ The main clinical manifestations are dyspnea, cough, and/or expectoration. Compared with other diseases, the incidence of COPD comorbidities is high, and the mortality rate also increases when there are comorbidities.^[2]^ In terms of treatment, COPD has no specific radical cure drugs and methods so far, and it is mainly divided into 3 stages: early intervention, stable treatment, and acute exacerbation treatment according to the progress of the disease. First of all, smoking cessation is the first and most important step in the treatment of COPD. Smoking cessation is considered to be effective in delaying the decline of patients lung function and improving patients clinical symptoms and quality of life.^[3]^ If respiratory failure occurs, it is recommended to use oxygen therapy, non-invasive, or invasive mechanical ventilation therapy.^[4]^ For very serious cases, if the quality of life is unacceptably low, surgical options include lung transplantation and lung volume reduction.^[5]^ In addition, in recent years, the application of pulmonary rehabilitation training in the treatment of COPD has gradually increased. Studies have shown that pulmonary rehabilitation exercises have good safety and effectiveness in the acute exacerbation or stable phase of COPD.^[6,7]^ Drug treatments include bronchodilators, expectorants, antibiotics, glucocorticoids, etc., especially the use of antibiotics in the treatment of COPD, and Studies have confirmed that long-term low-dose use of macrolides can significantly reduce the frequency of acute exacerbations of COPD,^[8]^ but long-term antibiotics have side effects such as drug resistance and fungal infection.^[9]^ In addition, the side effects of anticholinergic drugs, β2 receptor agonists, glucocorticoids and other drugs have also been confirmed and reported.^[10–12]^ Traditional Chinese medicine has a long history of treating COPD. In ancient China, COPD was named “Lung Swelling”, and many prescriptions were formulated to treat COPD. MSD is one of the main prescriptions for the treatment of COPD. It is a traditional Chinese medicine formula developed by Zhongjing Zhang, a famous doctor in the Eastern Han Dynasty, Zhongjing Zhang was revered as a medical saint by later generations. He is one of the most outstanding medical scientists in history of our country. MSD is composed of *Ephedra*, *Almonds*, *Gypsum,* and *Licorice*. The specific ratio of *Ephedra* and *Gypsum* is 1:2. This prescription has the effect of clearing the lungs and reaching lung qi.

*Ephedra*, the herb with pungent flavor, bitter flavor and mildness property, enters into the channels of lungs and bladder, and functions on sweating and dispelling cold, dispelling the lungs and relieving asthma, and relieving swelling. *Almonds*, the herb with bitter flavor and mildness property, enters into the channels of lungs and large intestines, and functions on lowering qi, relieving cough and asthma, and moistening the intestines. *Gypsum*, the herb with sweet flavor, pungent flavor, and cold property, enters into the channels of lungs and stomach, and functions on clearing away heat, purging fire, and quenching thirst. In the prescription, *Ephedra* and *Gypsum* are often used together as a fixed combination. *Licorice*, the herb with sweet taste flavor and peace property, enters into the channels of heart, lung, spleen, and stomach meridians, and functions on invigorating the spleen and qi, clearing heat and detoxification, eliminating phlegm and relieving cough, and relieving pain, and reconciling various drugs.

From the perspective of pharmacological research, the ephedrine, one of the main ingredients of *Ephedra*, has a relatively long-lasting antispasmodic effect on bronchial smooth muscle.^[13]^ The main component of *Almonds*, amygdalin, is the decomposition product of hydrocyanic acid in the body, which has a certain inhibitory effect on the respiratory center.^[14]^*Gypsum* decoction has an inhibitory effect on white blood cell pyrogenic fever and can also improve cellular immune function.^[15]^ Glycyrrhetinic acid has been proven to inhibit the proliferation of bronchial smooth muscle cells,^[16]^ and *Licorice* can promote the secretion of the throat and bronchi, and has an expectorant effect.^[17]^

A large number of experimental studies have confirmed the good effect of MSD in the treatment of COPD. However, we have not found a systematic study on the effectiveness and safety of MSD in the treatment of COPD. Therefore, we will adopt meta-analysis method to systematically evaluate the efficacy and safety of MSD in the treatment of COPD, and provide strong evidence-based medicine support for its clinical application.

## 2 Methods

### 2.1 Study registration

The program is reported in accordance with the Cochrane Handbook for Systematic Reviews of Interventions Version 5.1.0^[18]^ and the Preferred Reporting Project for Systematic Reviews and Meta-Analysis Programs (PRISMA-P) 2015 Statement.^[19]^ It has been registered on the Open Science Framework (OSF) platform (https://osf.io/azkuq), the registration number: DOI 10.17605/OSF.IO/H5UNB.

### 2.2 Ethics and dissemination

All analyses in this study are based on previously published studies, so this study does not require ethical approval or patient consent. We will disseminate our findings electronically or in print by publishing results in peer-reviewed journals.

### 2.3 Selection criteria

#### 2.3.1 Type of study design

This study only included RCTs, and non-randomized controlled trials were not included. Those with unclear diagnosis, inaccurate experimental protocol, incomplete, and accurate experimental data will be excluded.

#### 2.3.2 Type of participants

COPD patients who meet the diagnostic criteria of the Global Strategy for the Diagnosis, Management and Prevention of Chronic Obstructive Pulmonary Disease(2020 Report)^[1]^ have no restrictions on the gender, age, race, severity and duration of the disease, and patients with severe organ dysfunction diseases, Such as cancer, heart disease, liver disease or kidney disease, will be excluded from this study.

#### 2.3.3 Type of interventions

In the experimental group, use the original MSD or the modified MSD, but it must contain *Ephedra*, *Almonds,* and *Gypsum*, and the ratio of *Ephedra* to *Gypsum* is 1:2, and it is the only treatment method in the experimental group Or combined with other conventional therapies. The improved formula must comply with the “Jun-Chen-Zuo-Shi” principle of Chinese herbal formula. There are no restrictions on the dosage form, frequency, and time of decoction, but non-orally administered methods such as acupoint injection and intravenous infusion are not included. Control group: conventional medical treatment, such as antibiotics, bronchodilators, glucocorticoids, etc., health education is also included, but no form of Chinese medicine treatment is allowed.

#### 2.3.4 Type of outcome measures

##### 2.3.4.1 TCM syndrome points

According to the Guiding Principles for Clinical Research of New Chinese Medicines, the TCM syndrome score is determined as one of the main indicators. Judging criteria: If the patients related symptoms disappear or the symptoms are stable, the TCM symptom score reduction >75% is considered to be effective; the patients clinical symptoms are significantly improved, and the TCM symptom score reduction of 35% to 75% is considered effective; and Failure to meet the above standards is considered invalid.

##### 2.3.4.2 Pulmonary function indicators

Including the predicted value of forced end-expiratory volume in the first second (FEV1), forced vital capacity (FVC), and FEV1/FVC ratio.

##### 2.3.4.3 Other indicators

Serum inflammatory factors, including: IL-6, IL-8, CRP, and TNF-α.

Blood gas indicators, including: arterial partial pressure of carbon dioxide (PaCO_2_), arterial partial pressure of oxygen (PaO_2_), arterial oxygen saturation (SaO_2_).

##### 2.3.4.4 Safety outcomes

Occurrence of toxic reactions, idiosyncratic reactions, side effects, allergies, etc.

### 2.4 Search strategy

#### 2.4.1 Electronic search

Main information resource databases: PubMed, The Cochrane Central Register of Controlled Trials (CENTRAL), EMbase, China National Knowledge Infrastructure (CNKI), Chinese Biomedical Database(CBM), VIP Database, and Wanfang database. The search time limit is from the date of building the database to October 30, 2020. Keywords include: “chronic obstructive pulmonary disease”, “COPD”, “MSD”, and so on. A sample of PubMed search strategy is shown in Table 1.

**Table 1:** Search strategy of the PubMed.

Number	Search terms
#1	Chronic obstructive pulmonary disease [Mesh]
#2	Emphysema[Title/Abstract] OR Chronic lung disease[Title/Abstract]
#3	#1 OR #2
#4	Maxing Shigan [Title/Abstract]
#5	Decoction[Title/Abstract]
#6	#4 AND #5
#7	randomized controlled trial[Publication Type]
#8	controlled clinical trial[Publication Type]
#9	randomized[Title/Abstract]
#10	randomly[Title/Abstract]
#11	#10 OR #11 OR #12 OR #13
#12	#3 AND #6 AND #11

#### 2.4.2 Other search strategies

Manual search, including conference papers, searched literature references, and search and follow up research randomized controlled trials in clinical trial registration institutions such as the National Research Register, Meta-register of Controlled Trials, and China Clinical Trials Registration Platform.

### 2.5 Data collection and analysis

#### 2.5.1 Literature screening and data extraction

Import all search results into Endnote X9 for classification and sorting, and duplicate documents will be deleted. Two researchers (Jinyun Chen, Chunrong Wang) will independently read the titles and abstracts of the literature, and screen the literature based on the inclusion criteria. For any potential related research, we will download and read the full text, and decide to include or exclude it through discussions with other researchers.

**Figure 1:**
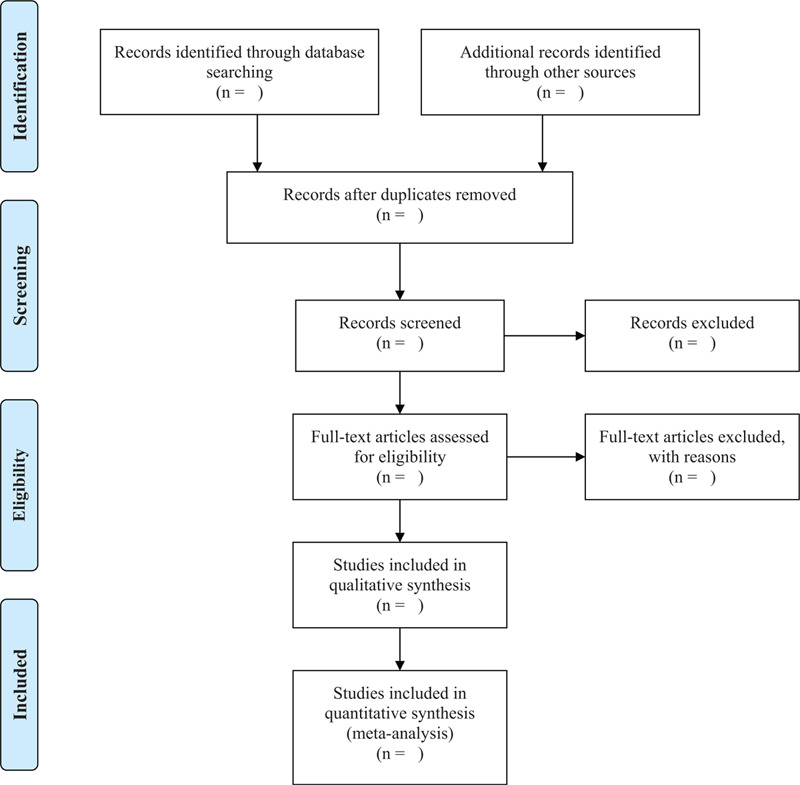
PRISMA flow diagram of the study selection process.

According to the inclusion criteria, the results of the included studies and all valuable information are correctly collected and recorded. Two researchers completed this work independently and reviewed each other. Data extraction includes 5 aspects: basic research information (title, journal, research ID number, author, and contact information, etc.), research method (research design, random unit, random method, etc.), observation object (age, gender, Sample size, etc.), intervention measures (dose, administration time, treatment course information, etc.), measurement indicators (measurement indicators and time points for judgment, judgment indicators, measurement units, etc.). If the data is missing, we will try to contact the original author. If no available data is available, we will exclude the study. Similarly, if the data is disputed, we will discuss with other researchers to decide (Fig. 1).

#### 2.5.2 Quality evaluation on methodology

The methodological quality of all qualified randomized controlled trials was evaluated according to Cochrane Handbook 5.2.0.^[20]^ There are 7 domains, and each item should be judged as 3 “low risk” deviations, “high risk” deviations, and “unclear” according to the quality classification standards. If there is a difference, it will be decided through collective consultation.

#### 2.5.3 Statistical analysis

The meta-analysis will be performed using RevMan 5.0 software provided by the Cochrane Collaboration Center. Binary variables and continuous variables are included in the results of interest. We will use a fixed or random effects model with a 95% confidence interval for the risk rate to analyze binary variables, and use the mean difference to evaluate the difference in continuous results between groups.

#### 2.5.4 Tests for heterogeneity

The tests for heterogeneity is a method used to test whether the statistics of multiple identical studies are heterogeneous. The heterogeneity of the included studies is measured by *I*^2^. When *I*^2^ is greater than or equal to 50%, the heterogeneity will be considered large. For this reason, we will use a random effects model to analyze the data. If *I*^2^ is less than 50%, it can be considered that multiple studies of the same kind are homogenous, and the fixed effects model can be used to analyze the data.

#### 2.5.5 Sensitivity analysis

To determine the stability and reliability of the summary results, a sensitivity analysis will be performed. Exclude some studies that are vague and less rigorous in design.

#### 2.5.6 Subgroup analysis

Subgroup analysis is to explore whether the results of the study are different due to the existence of some factors that may affect the prognosis. These factors include herbal dosage, disease duration, intervention measures, etc. When there is enough research, we will conduct subgroup analysis to explore the source of heterogeneity.

#### 2.5.7 Assessment of reporting bias

If more than 10 studies are included, we will make a funnel chart based on the data of the included studies to assess the deviation. When the funnel chart is asymmetrical, indicating that there may be publication bias, we will discuss the source and explain the possible causes of the bias.

#### 2.5.8 Grading the quality of evidence

In order to rate the quality of evidence, and to understand the actual situation of the evidence rating, so as to analyze the possible problems, the recommendation evaluation, development, and evaluation grade system will be used to evaluate the evidence quality.^[21]^ According to the recommendations of the GRADE working group, the evaluation of the evidence quality of the key outcome indicators can be divided into 4 levels: high (++++), medium (++), low (++), and very low (+).

## 3 Discussions

COPD is a common disease of the respiratory system, and its morbidity and mortality are increasing year by year. It has brought serious economic and social burdens to patients families and the country. Traditional Chinese medicine has its unique advantages in the treatment of COPD, because it has lower economic costs and less pain, and there are few reports on the side effects of traditional Chinese medicine in the treatment of COPD. At present, many clinical studies have confirmed the clinical efficacy of MSD in the treatment of COPD, but there is no comprehensive systematic review to provide sufficient evidence for this treatment. This systematic review and meta-analysis research program aims to evaluate the efficacy and safety of MSD in the treatment of COPD. I hope to provide COPD patients with more diverse treatment options and inspire more peer experts and doctors to carry out as many related studies as possible in the future. In addition, this study may have some limitations, because different ages, genders, races, drug formulations, dosages, and treatment courses can lead to heterogeneity.

## Author contributions

Jinyun Chen and Chunrong Wang made the same contribution to the research and design, and wrote the original draft of the protocol. Jinyun Chen has developed a search strategy. Jinyun Chen, Chunrong Wang and Min Xiong will conduct literature retrieval and collation. Jinyun Chen, Chunrong Wang, and Qilin Shen will evaluate the risk of bias in the literature. Data analysis and article writing will be done by Jinyun Chen, Chunrong Wang. Qilin Shen, as the corresponding author, will be responsible for overseeing every process of the audit review to control the quality of the study. All the authors have approved the publication of the protocol.

**Data curation:** Jinyun Chen, Chunrong Wang.

**Formal analysis:** Jinyun Chen, Chunrong Wang.

**Funding acquisition:** Min Xiong.

**Methodology:** Chunrong Wang.

**Project administration:** Jinyun Chen, Qilin Shen.

**Resources:** Min Xiong.

**Software:** Jinyun Chen.

**Writing – original draft:** Jinyun Chen, Chunrong Wang.

**Writing – review & editing:** Qilin Shen.
